# The effects of microbial fertilizer based *Aspergillus brunneoviolaceus* HZ23 on pakchoi growth, soil properties, rhizosphere bacterial community structure, and metabolites in newly reclaimed land

**DOI:** 10.3389/fmicb.2023.1091380

**Published:** 2023-02-06

**Authors:** Xuqing Li, Dingyi Li, Yugen Jiang, Jun Xu, Xiaoxu Ren, Ya Zhang, Hong Wang, Qiujun Lu, Jianli Yan, Temoor Ahmed, Bin Li, Kai Guo

**Affiliations:** ^1^Institute of Vegetable, Hangzhou Academy of Agricultural Sciences, Hangzhou, China; ^2^Department of Biological Environment, Material and Environmental CollegeShanxi Jinzhong Institute of Technology, Jinzhong, China; ^3^Agricultural Technology Extension Center of Fuyang District, Hangzhou, China; ^4^Hangzhou Agricultural and Rural Affairs Guarantee Center, Hangzhou, China; ^5^Institute of Biotechnology, Zhejiang University, Hangzhou, China; ^6^School of Forestry and Biotechnology, Zhejiang A&F University, Hangzhou, China

**Keywords:** pakchoi, newly reclaimed land, MF-HZ23, commercial compound fertilizer, soil property, bacterial community, metabonomics

## Abstract

**Introduction:**

Pakchoi is an important leafy vegetable in China. Due to industrialization and urbanization, pakchoi has been cultivated in newly reclaimed mountainous lands in Zhejiang Province, China in recent years. However, immature soil is not suitable for plant growth and needs to be modified by the application of different organic fertilizer or microbial fertilizer based plant-growth-promoting microbe. In 2021, a high efficient plant-growth-promoting fungi (PGPF; *Aspergillus brunneoviolaceus* HZ23) was obtained from newly reclaimed land of Zhejiang Province, China. In order to valuate microbial fertilizer based A. brunneoviolaceus HZ23 (MF-HZ23) on pakchoi growth in immature soil, we investigated the effect of MF-HZ23 on soil properties, rhizosphere bacterial community structure, and metabolites of pakchoi rhizosphere soil samples.

**Methods:**

The field experiment (four treatments, MF-HZ23, MF-ZH23 + CCF, CCF and the control) was completely randomly designed and carried out on newly reclaimed land in Yangqingmiao Village of Fuyang district, Hangzhou City, Zhejiang Province, China. In order to evaluate the influence of microbial fertilizer based *A. brunneoviolaceus* HZ23 on pakchoi in the newly reclaimed land, the number of pakchoi leaves, total fresh and dry weight of the seedlings was counted. In addition, the soil properties, including the pH, OMC, total N, AHN, available P, the genome sequencing, and metabolomics assay were also detected.

**Results:**

The results revealed a significant difference between MF-HZ23 and the control in soil properties, bacterial community structure, and metabolites. Indeed, compared with the control, MF-HZ23 caused 30.66, 71.43, 47.31, 135.84, and 2099.90% increase in the soil pH, organic matter contents (OMC), total nitrogen (N), alkaline hydrolysis nitrogen (AHN), and available phosphorus (P), respectively. Meanwhile, MF-HZ23 caused 50.78, 317.47, and 34.40% increase in the relative abundance of *Proteobacteria*, *Bacteroidota*, and *Verrucomicrobiota* and 75.55, 23.27, 69.25, 45.88, 53.42, and 72.44% reduction in the relative abundance of *Acidobacteriota, Actinobacteriota, Chloroflexi, Planctomycetota, Patescibacteria*, and *WPS-2*, respectively, compared with the control based on 16S amplicon sequencing of soil bacteria. Furthermore, redundancy discriminant analysis (RDA) of bacterial communities and soil properties indicated that the main variables of bacterial communities included available P, AHN, pH, OMC, and total N. In addition, non-targeted metabolomics techniques (UHPLC–MS analysis) revealed that MF-HZ23 resulted in a great change in the kinds of metabolites in the rhizosphere soil. Indeed, in MF-HZ23 and the control group, there were six differentially expressed metabolites (DEMs) belong to organoheterocyclic compounds, organic acids and derivatives, organic nitrogen compounds, and these six DEMs were significantly positively correlated with 23 genus of bacteria, which showed complicated interactions between bacteria and DEMs in pakchoi rhizosphere soil.

**Conclutions:**

Overall, the results of this study revealed significant modification in physical, chemical, and biological properties of pakchoi soil. Microbial fertilizer based PGPF *A. brunneoviolaceus* HZ23 (MF-HZ23) can be used as a good amendment for newly reclaimed land.

## 1. Introduction

Pakchoi (*Brassica chinensis* L.) is an important leafy vegetable, which was largely consumed in China in recent years due to the richness in vitamin C and minerals (Hanson et al., 2009; Wang et al., 2014; Ren et al., 2020). However, with industrialization and urbanization, a large amount of cultivated land resources has been occupied, which has become an obstacle to the development of agriculture (Ghose, 2014). To meet the demand for cultivated land, pakchoi has been cultivated in newly reclaimed mountainous lands in Zhejiang Province, China in recent years. However, in most situations, newly reclaimed land is acidic and poor in nutrients with high gravel content, which lead to it not suitable for plant growth (Li et al., 2021a,b,c). Therefore, to develop the production of mountainous pakchoi in newly reclaimed land in Zhejiang Province, China, it is very necessary to find effective measures to improve the quality of the immature soil.

It is well known that the quality of soil is highly associated with physical, chemical, and biological properties (Li et al., 2021a). Previous research showed that the application of organic fertilizer, such as sheep manure, biogas liquid, mushroom residue, pig manure, and mushroom residue organic fertilizer, and so on, could not only modify physical and chemical properties of soil but also had a great influence on microbial communities in newly reclaimed land (Li et al., 2021a, 2022a,b). In addition, research also showed that the application of microbial fertilizer based soil microbe, such as Tuzangjin microbial fertilizer, carbonergic microbial agent, and seaweed microbial fertilizer, played an important role in soil ecosystems and nutrient transformation (Li et al., 2022c). Whereas microbial-organic fertilizer composed of specific functional microbes and organic fertilizer (usually animal residues), could improve soil physical and chemical properties (Shang et al., 2020), enrich organic matter and balance nutrient levels (Zubair et al., 2020), regulate the structure and function of the microbial community (Li et al., 2014), promote plant growth, improve crop yield (Wang et al., 2020), and prevent plant disease (Tao et al., 2020). Research showed that microbes were very important in agriculture ecosystems by changing microbial communities to maintain soil fertility and promote plant growth (Ren et al., 2021b). Host plants have been reported to form an intimate association with microbes, which were attracted by plant root exudates (Vacheron et al., 2013). On the other hand, microbes can promote plant growth by transforming, solubilizing, and mobilizing soil nutrients. In addition, organic acids, sugars, and other soil metabolites also played an important role in the rhizosphere soil environment by playing various roles (toxic or beneficial effect) in plant and microbe interaction (Zhang et al., 2015; Zhao et al., 2019; Chen et al., 2021). In other words, environmental factors, such as nutrients and microbes, always influence plant growth (Chen et al., 2021). Therefore, more attention should be paid on the relationship of soil, microbes, and plant from several view angles.

Plant growth promoting fungi (PGPF) is receiving more and more attention in recent days (Hossain and Sultana, 2020). Varieties of PGPF belong to genera *Penicillium*, *Fusarium*, and *Phoma* have been studied over the last decades (Hyakumachi, 1994; Li et al., 2021b). Research showed that PGPF could modulate plant growth and suppress abiotic stresses through a wide complex mechanism (Hossain et al., 2017), including phosphorus (P) solubilization, siderophores, extracellular enzymes, indoleacetic acid (IAA), and volatile organic compounds production, and so on. These wide arrays of interconnected mechanisms helped PGPF maintaining rhizosphere competence and stability in host performance. But compared to the large number of PGPF identified in the laboratory, only a few of them were in agricultural practice worldwide, because the inconsistent performance of PGPF under field condition limited the application of PGPF (Hossain and Sultana, 2020). In 2021, four high efficient PGPF were obtained in our previous study by the screening of P solubilization, siderophore, and IAA production from newly reclaimed land in Zhejiang Province, China, and results of pot experiments showed that the four isolates except HZ123 significantly promoted eggplant seedling growth at different levels under greenhouse conditions. However, it is still unclear whether these PGPF have a promotion effect on the growth of pakchoi plants in newly reclaimed land (under field conditions).

We hypothesized that the microbial fertilizer based *A. brunneoviolaceus* HZ23 (MF-HZ23) has a beneficial effect in the growth of pakchoi by improving the soil quality of newly reclaimed land. Thus, the aim of this study was to evaluate the effect of MF-HZ23 on pakchoi growth, rhizosphere soil properties, bacterial community structure, and metabolites in newly reclaimed land. In addition, we examined the correlation between soil properties and the bacterial community structure, the correlation between DEMs and related bacteria. This study provides a scientific basis to develop an effective measure to improve the soil quality of newly reclaimed land, and increase the production of pakchoi in the future.

## 2. Materials and methods

### 2.1. Experimental design

The field experiment was carried out on newly reclaimed land in Yangqingmiao Village (30°3′57″N, 119°51′51″E, 53 m above sea level) of Fuyang district, Hangzhou City, Zhejiang Province, China. The soil type is acrisols (acidic red soil), based on the soil classification system of the FAO-UNESCO, and the top 20 cm soil had a pH of 4.96, with 0.67% of OMC, 0.72 g/kg of total N, 34.67 mg/kg of AHN, and 37.03 mg/kg of available P. In detail, before the pakchoi was planted, about 1.0 kg of fresh soil (0–20 cm) of the test filed was collected using the quartering method (Campos-M and Campos-C, 2017). After air-drying at room temperature, the soil properties were measured. Each treatment consisted of three replicates.

The experiment consisted of four different treatments through the application of microbial and chemical fertilizers to newly reclaimed land. The microbial fertilizer (MF-HZ23) was provided by Hangzhou Academy of Agricultural Sciences, Hangzhou, China, which contained sheep manure, mushroom residue, and the PGPF *A. brunneoviolaceus* HZ23 isolated from newly reclaimed land in Yaolin town, Tonglu city, Zhejiang Province, China (Li et al., 2021b), and the final concentration of HZ23 in MF-HZ23 was 10^8^ spores/g. The chemical compound fertilizer (CCF, N-P-K, 15-15-15) was provided by Henan Xinlianxin Chemical Industry Group Co., Ltd., Xinxiang, China. The treatment without any fertilizer or microbe was applied as the control. The information of each treatment used in this study was shown in Table 1: MF-HZ23 at 3.00 kg/m^2^ (T1), MF-HZ23 at 3.00 kg/m^2^ plus CCF at 0.02 kg/m^2^ (T2), CCF at 0.04 kg/m^2^ (T3), and without any MF-HZ23 or CCF (control).

**Table 1:** The information of each treatment used in this study.

Treatments	Amendments
T1	MF-HZ23 (3.00 kg/m^2^)
T2	MF-HZ23 (3.00 kg/m^2^) + CCF (0.02 kg/m^2^)
T3	CCF (0.04 kg/m^2^)
Control	*–*

MF-HZ23, microbial fertilizer based *A. brunneoviolaceus* HZ23, pH 7.2, organic matter contents (OMC) ≥ 68.75%, NPK content 7.8%; CCF, chemical compound fertilizer, pH 4.5, NPK content 45.0%.

The field experiment was completely randomly designed in the study and carried out from 20 October to 14 December in 2021. The area of each plot was 34 m^2^, and the length and width of the plot were 200 and 170 cm, respectively, and the planting density of pakchoi was 25 cm × 25 cm. On 20 October, the top 0–20 cm soil of the experimental field was mixed with different fertilizers (MF-HZ23, MF-HZ23 plus CCF, CCF, and without any MF-HZ23 or CCF as the control) before planting, and then the seedlings of pakchoi (cultivar “Heitiane 2,” provided by Qingdao Shenrong Agricultural Development Co., Ltd., Qingdao, China) were planted in the above-mentioned newly reclaimed land. In detail, the seeds of pakchoi were sown into 50 cells of seed-growing trays containing newly reclaimed land soil on 29 September and moved to a greenhouse with a relative humidity level of 65–75% and a temperature of 25°C for 3 weeks. The number of leaves was counted after 3 weeks of planting. The seedlings were harvested after 55 days of planting, and the total fresh and dry weight were counted. Each treatment had three replicates.

### 2.2. Measure of pakchoi parameters and soil properties

In order to evaluate the influence of microbial fertilizer based *A. brunneoviolaceus* HZ23 on the biomass of pakchoi in the newly reclaimed land, the number of pakchoi leaves was counted after 3 weeks of planting, and the total fresh weight and dry weight of pakchoi was measured after 55 days of planting by a digital scale (TCS-50, Shanghai hento Industrial Co., Ltd., Shanghai, China). In detail, the pakchoi was dug up using hoes from the soil, and the fresh weights were measured after removing the soil from the root by tap water. Dry weights were measured by drying pakchoi organs in an oven at 65°C for 3 days. The growth promotion efficacy (GPE%) was assessed using the following formula: GPE% = (treatment – control)/control × 100%.

The soil properties, including the pH, OMC, total N, AHN, and available P, were detected as described in Baran et al. (2019); Dodor and Tabatabai (2020). In detail, when pakchoi was collected, about 1.0 kg of fresh rhizosphere soil (5–20 cm) of each plot was sampled using the quartering method and a shovel with a scale (Campos-M and Campos-C, 2017). After passed through a 0.45-mm sieve to remove fine roots and debris and dried at room temperature, the soil properties were measured. Briefly, the soil pH was measured at a soil/distilled water suspension ratio of 1:5 (g/ml) with a pH meter (FE28, MettlerToledo, Zurich, Switzerland); the content of organic matter was determined by the K_2_Cr_2_O_7_ oxidation external heating method; the content of total N was determined using an automatic Kjeldahl distillation-titration unit; the AHN was determined by 1 M KOH or NaOH; the content of available P was determined by hydrochloric acid–ammonium fluoride extraction molybdenum–antimony anti–colorimetry. All the treatments had three replicates.

### 2.3. Genome sequencing

When pakchoi was collected on 14 December in 2021, 20 g rhizosphere soil of pakchoi was sampled of each plot and stored at −40°C. The DNA extraction of the soil samples was extracted using the E.Z.N.ATM Mag–Bind Soil DNA Kit (OMEGA, Norcross, GA, United States) following the manufacturer′s instructions. The quality of extracted DNA was determined using a NanoDrop (ND-1000) spectrophotometer (ThermoFisher Scientific, United States).

The PCR amplification for the V3-V4 region of pakchoi rhizosphere bacterial 16S rRNA genes was carried out using the universal primers 341F (5′–CCTACGGGNGGCWGCAG–3′) and 805R (5′–GACTACHVGGGTATCTAATCC–3′; Wu et al., 2015). The components of the PCR included 12 μl ddH_2_O, 15 μl 2 × Hieff® Robust PCR Master Mix, 1 μl DNA template, and 1 μl (10 μM) each universal primer. The PCR thermal cycle consisted of an initial denaturation of 4 min at 98°C, followed by 25 cycles of denaturation at 98°C for 30 s, annealing at 53°C for 30 s, expansion at 72°C for 45 s, and finally an extension of 8 min at 72°C. The PCR amplicons were purified with Vazyme VAHTSTM DNA clean beads (Vazyme, Nanjing, China). Afterward, amplicons with equal amounts were pooled and 2 × 250 bp pair-end sequencing was accomplished through the Illumina MiSeq system (Wuhan Bena Technology Co., Ltd., Wuhan, China).

The bioinformatics analysis of the microbe was accomplished as described in our previous study (Jiang et al., 2022; Li et al., 2022c). In detail, to ensure data quality, low-quality reads (average quality score < 20) were removed by preprocessing raw sequencing reads using Trimmomatic (v0.39; Bolger et al., 2014), and primers were trimmed with the Cutadapt (v3.5; Martin, 2011). Reads were quality filtered, denoised, merged, chimera filtered using DADA2 (Callahan et al., 2016). Then clean reads were analyzed using the “classify-sklearn” package in the QIIME2 (v2018.08) to assign taxonomy to amplicon sequence variants (ASVs) against the SILVA Release 138 Database (Bokulich et al., 2018).

### 2.4. Metabolomics assay

When pakchoi was collected on 14 December 2021, 10 g rhizosphere soil of pakchoi was sampled of each plot and stored at −80°C. After thawed at 4°C, 1 g of soil sample was extracted in 2:2:1 precooled methanol:acetonitrile:H_2_O (v/v/v), and analyzed by ultra-high performance liquid chromatography-mass spectrometry (UHPLC-MS) *via* a Thermo Exactive mass spectrometer (Q-Exactive HF MS, Thermo, Waltham, MA, United States) with ESI. In detail, the mixture was vortexed and ultrasonicated for 30 min, placed at −20°C for 10 min, and centrifuged for 20 min (14,000 rpm, 4°C). The supernatant was placed into a new 2 ml centrifuge tube and freeze-dried. For UHPLC–MS metabolomics analysis, the dried powder was re-dissolved into 100 μl 1:1 acetonitrile:H_2_O (v/v), vortexed, and centrifuged for 15 min (14,000 rpm, 4°C), then the supernatant was transferred to UHPLC glass vials. The UHPLC–MS analysis conditions were set as follows: chromatographic column: waters ACQUITY UPLC BEH Amide (1.7 μm, 2.1 mm × 100 mm); mobile phase A: 25 mM ammonium acetate and 25 mM ammonium hydroxide in water, mobile phase B: acetonitrile; gradient program: 95% B at 0–0.5 min, 95–65% B at 0.5–7 min, 65–40% B at 7–8 min, 40% B at 8–9 min, 40–95% B at 9–9.1 min, 95% B at 9.1–12 min; column temperature: 25°C, flow rate: 0.5 ml/min, and sample size: 2 μl. The ESI source conditions were set as follows: ion source gas1 (GS1), 60 psi; ion source gas2 (GS2), 60 psi; curtain gas (CUR), 30 psi; temperature, 600°C; and ion spray voltage floating, ±5,500 V. Samples were run in both positive and negative ionization mode, and MS data were collected in profile mode over the mass range of *m/z* 70–1,200. The repeatability of the entire analysis process was examined by inserting one quality control (QC) sample. The QC samples were prepared by pooling and combining 10 μl of each sample. All the treatments had three replicates. The obtained data in this study were compared with the in-house database (Shanghai Applied Protein Technology; Luo et al., 2017; Gu et al., 2018), and the obtained metabolite information was searched for the Kyoto Encyclopedia of Genes and Genomes (KEGG) database.

### 2.5. Statistical analysis

The SPSS 16.0 software (SPSS Inc., Chicago, IL, United States) was used to calculate the significance test (*p* < 0.05) of the main treatments and their interaction through an analysis of variance (ANOVA) after testing for normality and variance homogeneity. The ASVs and alpha diversity indices including Chao1 and Shannon index, was analyzed by Origin (v2022) and visualized in the bar graphs. The analysis of beta diversity was carried out to observe the structural variation of rhizosphere soil microbe across samples with Bray-Curtis metrics, principal component analysis (PCA; Ramette, 2007). The significant differences of rhizosphere soil microbe between groups were tested by permutational multivariate ANOVA (PERMANOVA), with 999 permutations used to calculate *p* values (Dixon, 2003). Linear discriminant analysis effect size (LeFSe) was carried out by using default parameters to observe the differentially abundant taxa of rhizosphere soil microbe between groups (Segata et al., 2011). To investigate the impact of environmental factor (such as pH, OMC, total N, AHN, and available P) on bacterial community structure, redundancy discriminant analysis (RDA) was carried out using Origin (v2022). To investigate the effect of MF-HZ23 on the metabolites, orthogonal partial least-squares discriminant analysis (OPLS-DA) and volcano plot on different treatments were conducted with the MetaboAnalyst 4.0 platform. The thresholds for screening significant DEMs were set as follows: fold change (FC) > 1.5 or < 0.67, variable importance in the projection (VIP) > 1, and *p* < 0.05. To investigate the correlation between differential bacteria and DEMs in different treatment groups, a Spearman correlation coefficient among the high relative abundances of pakchoi rhizosphere soil bacteria (top 50 bacteria at genus level) and DEMs (the largest VIP value, *p* < 0.05) was measured by *p* < 0.05, Spearman’s coefficient *N* > 0.6 or < −0.6 (Hollander et al., 2008).

## 3. Results

### 3.1. Effects of MF-HZ23 on pakchoi production

**Figure 1:**
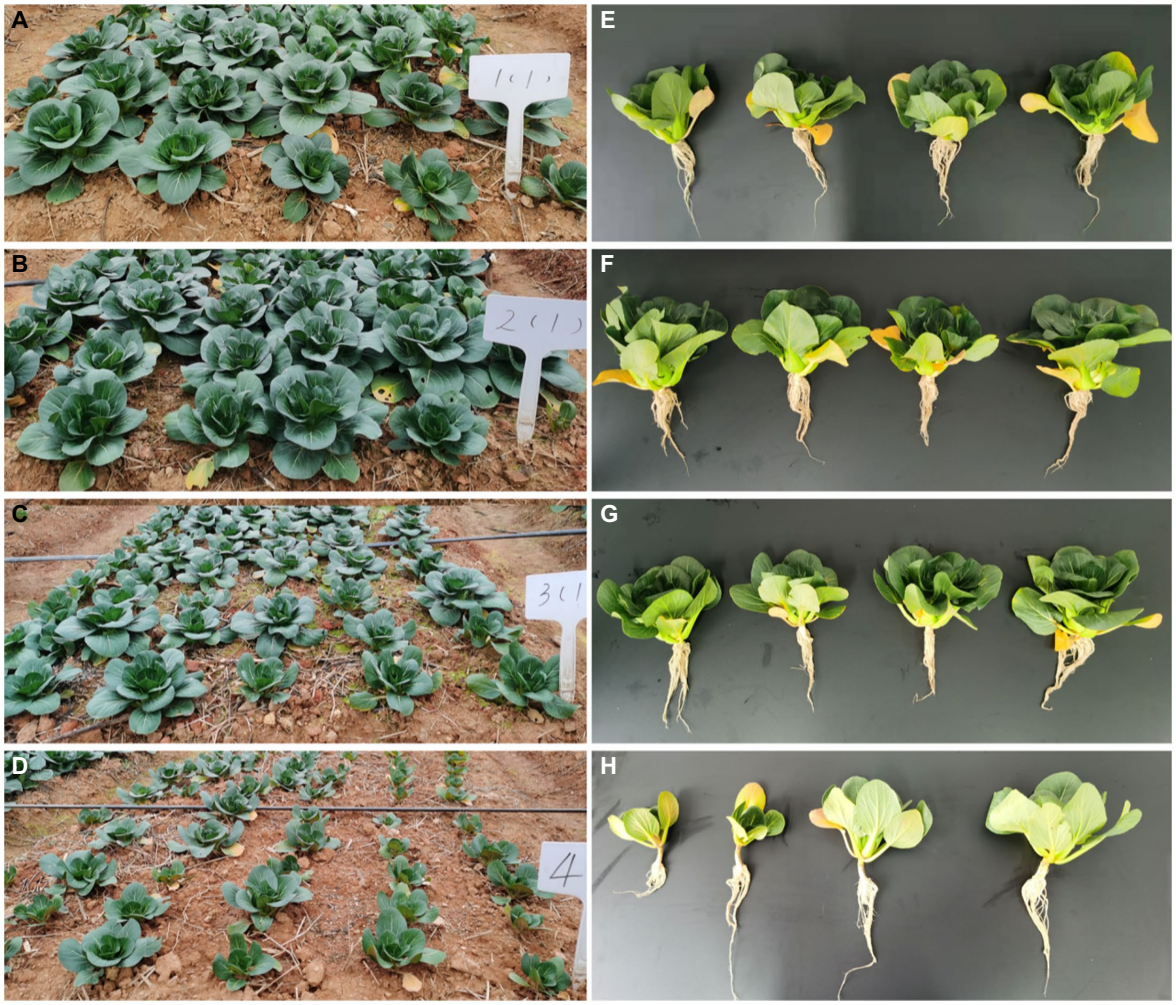
Effect of MF-HZ23 on the growth of pakchoi in the newly reclaimed filed. **(A,E)** MF-HZ23 at 3.00 kg/m^2^ (T1); **(B,F)** MF-HZ23 at 3.00 kg/m^2^ plus CCF at 0.02 kg/m^2^ (T2); **(C,G)** CCF at 0.04 kg/m^2^ (T3); and **(D,H)** control.

To evaluate the effect of MF*-*HZ23 on pakchoi production on newly reclaimed land, the leaf number of pakchoi was counted 3 weeks after planting, and the fresh and dry weight of pakchoi was measured about 55 days after planting when harvested. The results showed that MF-HZ23 significantly promoted pakchoi growth at different levels under field conditions. Based on the phenotypic observation, MF-HZ23 caused an obvious increase (81.82–103.03%) in leaf number and affected the biomass accumulation of pakchoi compared to the control (Figure 1; Table 2). Indeed, compared to the control, application of MF*-*HZ23 (T1) caused a 641.74 and 385.95% increase, while MF-HZ23 plus CCF (T2) resulted in a 1010.11 and 426.20% increase, in the fresh weight and dry weight of seedlings, respectively. Furthermore, the fresh weight and dry weight of pakchoi seedlings in MF-HZ23 (T1) is 1.79- and 1.94-fold, while MF-HZ23 plus CCF (T2) treatment is 2.68- and 2.10-fold greater than that of CCF treatment (T3), respectively. This indicated that MF-HZ23 generally had a greater effect on pakchoi growth compared to the control.

**Table 2:** Effects of MF-HZ23 on growth promotion of pakchoi.

Treatments	LN	TFW (g)	GPE%	TDW (g)	GPE%
T1	10.00 ± 0.89 ab	191.99 ± 4.79	641.74 b	22.01 ± 1.76	385.95 b
T2	11.17 ± 0.98 a	287.33 ± 4.45	1010.11 a	23.84 ± 2.04	426.20 a
T3	9.67 ± 0.82 b	107.29 ± 4.49	314.53 c	11.37 ± 0.88	151.04 c
Control	5.67 ± 0.84 c	25.88 ± 2.67	–	4.53 ± 0.87	–

LN, leaf number; TFW, total fresh weight; TDW, total dry weight; and GPE, growth promotion efficacy. Means are averages ± SD. Different lowercase letters within the same columns reveal the significance among different treatments (*p* < 0.05).

### 3.2. Effects of MF-HZ23 on soil pH, OMC, and nutrient elements

Results from this study indicated that the application of MF-HZ23 significantly raised the pH, OMC, total N, AHN, and available P of pakchoi rhizosphere soil (Table 3). In detail, compared with the control, MF-HZ23 (T1) and MF-HZ23 plus CCF (T2) caused a 30.66 and 10.08% increase, while CCF (T3) caused a 6.38% reduction in the soil pH, respectively. Furthermore, the soil OMC was significantly increased 71.43 and 26.19% by MF-HZ23 (T1) and MF-HZ23 plus CCF (T2), respectively, while reduced 19.05% by CCF (T3) compared to the control. Meanwhile, compared to the control, MF-HZ23 (T1) and MF-HZ23 plus CCF (T2) caused a significantly increase by 47.31 and 21.51%, while CCF (T3) caused a 4.30% reduction in total N, respectively. The content of AHN was significantly increased by MF-HZ23 (T1) and MF-HZ23 plus CCF (T2), with a 135.84 and 55.39% increase, respectively, but was reduced by CCF (T3), with a 1.35% reduction compared to the control. And compared to the control, MF-HZ23 (T1), MF-HZ23 plus CCF (T2) and CCF (T3) caused significantly increase in available P, which caused a 2099.90, 1183.47, and 26.19% increase, respectively.

**Table 3:** Effects of MF-HZ23 on the pH and chemical properties on pakchoi soil.

Treatments	pH	OMC (%)	Total N (g/kg)	AHN (mg/kg)	Available P (mg/kg)
T1	6.35 ± 0.43 a	1.44 ± 0.19 a	1.37 ± 0.15 a	95.80 ± 1.47 a	854.09 ± 3.38 a
T2	5.35 ± 0.10 b	1.06 ± 0.13 b	1.13 ± 0.11 b	63.12 ± 2.64 b	498.30 ± 4.38 b
T3	4.55 ± 0.06 c	0.68 ± 0.03 c	0.89 ± 0.02 c	40.07 ± 0.56 c	48.99 ± 2.64 c
Control	4.86 ± 0.09 c	0.84 ± 0.12 c	0.93 ± 0.12 c	40.62 ± 1.65 c	38.82 ± 1.25 d

OMC, organic matter contents; AHN, alkaline hydrolysis N. Means are averages ± SD. Different lowercase letters within the same columns reveal the significance among different treatments (*p* < 0.05).

### 3.3. The effect of MF-HZ23 in microbial community diversity

**Figure 2:**
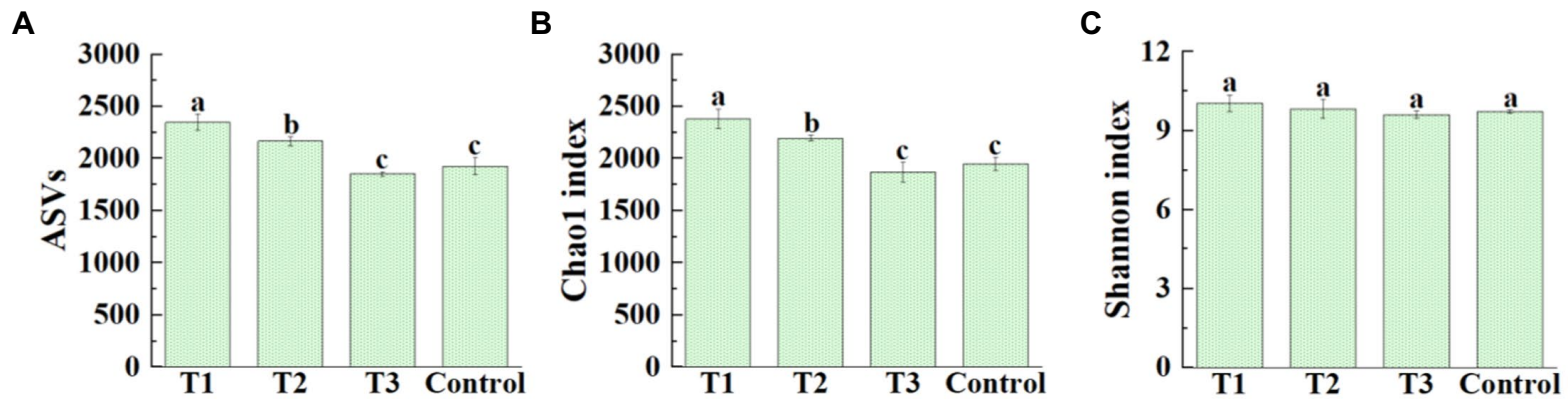
Effect of MF-HZ23 on the ASVs distribution **(A)**, Chao1 richness index **(B)**, and Shannon′s diversity index **(C)** of bacteria in pakchoi rhizosphere soil. T1: MF-HZ23 at 3.00 kg/m^2^; T2: MF-HZ23 at 3.00 kg/m^2^ plus CCF at 0.02 kg/m^2^; T3: CCF at 0.04 kg/m^2^; T4: control. Different lower case letters above columns indicate statistical differences (*p* < 0.05).

After original data quality-controlled, a total of 1,069,269 high-quality16S rRNA gene sequences were obtained from all samples of four different treatments. Among them, the high-quality sequences of each sample range from 60,540 to 108,257. A total of 24,859 bacterial ASVs were identified, and distribution of ASVs in four different treatments was shown in Figure 2A. The average number of bacterial ASVs was 2347.00 (2,283–2,434), 2165.00 (2,118–2,205), 1850.00 (1,826–1,868), 1924.33 (1,831–1,988) in MF-HZ23 (T1), MF-HZ23 plus CCF (T2), CCF (T3), and the control, respectively. Meanwhile, the richness index (Chao1) and diversity index (Shannon) was chosen to evaluate the alpha diversity of bacterial community (Figures 2B,C). The average Chao1 index was 2379.70 (2311.20–2491.61), 2193.89 (2162.07–2210.79), 1870.23 (1769.41–1951.98), 1949.19 (1901.00–2020.89), and the Shannon index was 10.06 (9.74–10.26), 9.84 (9.60–10.27), 9.62 (9.48–9.73), and 9.73 (9.68–9.79) in MF-HZ23 (T1), MF-HZ23 plus CCF (T2), CCF (T3), and the control, respectively. In general, the bacterial ASVs number was significantly increased (21.96 and 12.51%, respectively) by MF-HZ23 (T1), MF-HZ23 plus CCF (T2), and slightly reduced (3.86%) by CCF (T3) compared with the control, respectively (Figure 2A). The bacterial Chao1 index was significantly increased (22.09 and 12.55%, respectively) by MF-HZ23 (T1), MF-HZ23 plus CCF (T2), and slightly reduced (4.05%) by CCF (T3) compared with the control, respectively (Figure 2B), but no significant difference was observed in the Shannon index of bacterial community among all four different treatments (Figure 2C). Obviously, the bacterial richness and diversity was differentially affected by different fertilizers, and the application of MF-HZ23 could significantly increase the richness of bacteria in the rhizosphere soil of pakchoi in newly reclaimed land.

### 3.4. The effect of MF-HZ23 in soil microbial community structure

**Figure 3:**
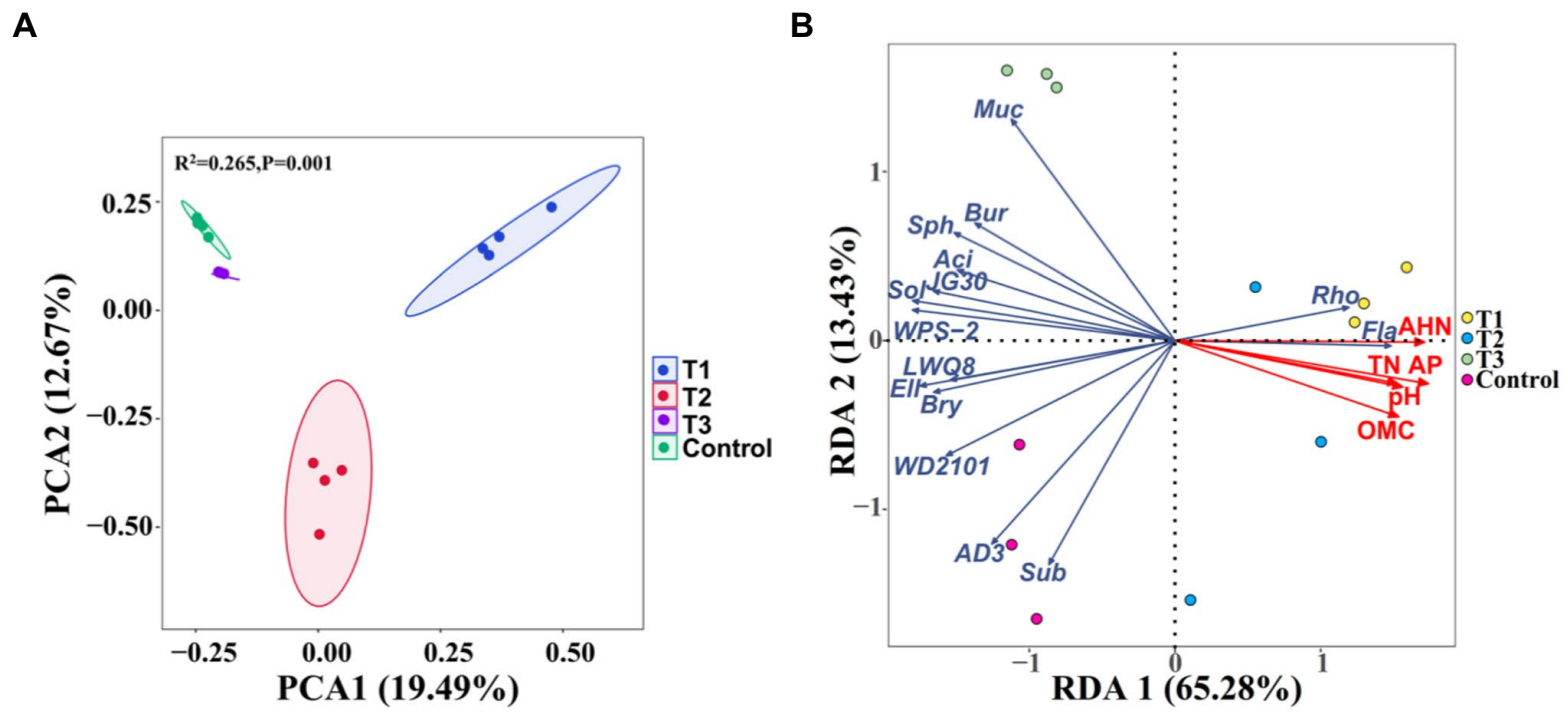
Principal component analysis (PCA) of the rhizosphere bacterial communities based on ASVs abundance **(A)**, and redundancy discriminant analysis (RDA) of the rhizosphere bacterial community compositions at genus levels with soil physicochemical properties **(B)**. Ellipses have been drawn for each treatment with a confidence limit of 0.95. Sph, Sphingomonas; Bur, Burkholderia; Fla, Flavobacterium; Rho, Rhodanobacter; Muc, Mucilaginibacter; Sol, Solibacter; Sub, Subgroup2; Bry, Bryobacter; Aci, Acidibacter; and Ell, Ellin6067. OMC, organic matter contain; TN, total N; AP, available P; and AHN, alkaline hydrolysis N. Arrows indicate the direction and magnitude of soil physicochemical properties (pH, OMC, total N, AP, and AHN) associated with the different bacterial genus. T1: MF-HZ23 at 3.00 kg/m^2^; T2: MF-HZ23 at 3.00 kg/m^2^ plus CCF at 0.02 kg/m^2^; T3: CCF at 0.04 kg/m^2^; and T4: control.

Principal component analysis (PCA) based on the Bray-Curtis distance was performed to further compare the effect of MF-HZ23 on the rhizosphere bacterial community (Figure 3A). The PCA analysis showed that the soil rhizosphere bacterial community of MF-HZ23 (T1), MF-HZ23 plus CCF (T2), CCF (T3), and the control formed four different groups, and there was no overlap among all four different treatments. The samples of all four different treatments were separated along the first axis (PERMANOVA, *p* < 0.05). The first axis explains 19.49% of the overall variation, and the second axis explains 12.67%. PERMANOVA analysis on samples of all four different treatments also showed that different fertilizers explained 26.5% of the variation (*p* = 0.001). Results showed that the bacterial community structure of the rhizosphere soil was significantly changed by different fertilizers including MF-HZ23.

**Figure 4:**
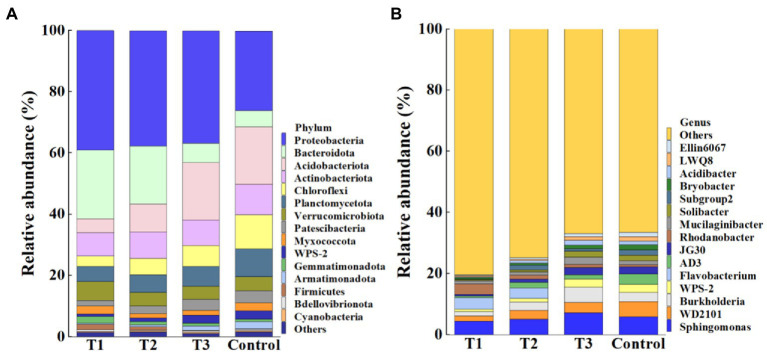
Relative abundance of bacteria at the phylum **(A)** and genus **(B)** level. T1: MF-HZ23 at 3.00 kg/m^2^; T2: MF-HZ23 at 3.00 kg/m^2^ plus CCF at 0.02 kg/m^2^; T3: CCF at 0.04 kg/m^2^; and T4: control.

Meanwhile, results showed that the application of MF-HZ23 resulted in a significant change in the composition of the bacterial community at the phylum (Figure 4A) and genus levels (Figure 4B) compared to the control. In detail, the top 15 phylum in the rhizosphere soil of pakchoi were selected to generate a relative abundance histogram, in which *Protebacteria*, *Bacteroidota*, *Acidobacteriota*, *Actinobacteriota*, *Chloroflexi*, *Planctomycetota*, *Verrucomicrobiota*, *Patescibacteria*, and *Myxococcota* were the main bacterial phylum with a relative abundance of 25.87–39.01, 5.36–22.40, 4.60–18.79, 7.58–9.88, 3.41–11.10, 4.90–9.06, 4.22–6.12, 1.87–4.02, and 1.37–2.63%, respectively (Figure 4A). Furthermore, the relative abundance histogram based on the top 15 species showed that *Sphingomonas*, *WD2101*, *Burkholderia*, and *Mucilaginibacter* were the main bacterial genes with a relative abundance of 4.47–7.23, 1.71–5.06, 1.45–4.98, and 1.07–2.41%, respectively (Figure 4B). Compared with that of the control, the relative abundance of *Sphingomonas* was reduced by 23.80 and 12.54% in MF-HZ23 (T1) and MF-HZ23 plus CCF (T2), but increased by 23.20% in CCF (T3), respectively. The relative abundance of *WD2101* was reduced by 66.18, 42.87, and 33.65% in MF-HZ23 (T1), MF-HZ23 plus CCF (T2), and CCF (T3), respectively. The relative abundance of *Burkholderia* was reduced by 50.67 and 6.68% in MF-HZ23 (T1) and MF-HZ23 plus CCF (T2), but increased by 69.99% in CCF (T3), respectively. The relative abundance of *Mucilaginibacter* was reduced by 18.90 and 21.42% in MF-HZ23 (T1) and MF-HZ23 plus CCF (T2), but increased by 76.41% in CCF (T3), respectively. Results indicate that the change in the number of specific bacteria in different treatments may be due to the different nutrients under different fertilizers conditions, and different fertilizers including MF-HZ23 could affect the abundance of bacteria in the rhizosphere soil of packchoi to regulate the composition of the bacterial community.

### 3.5. The effect of MF-HZ23 in the rhizosphere microbiome and biomarker

**Figure 5:**
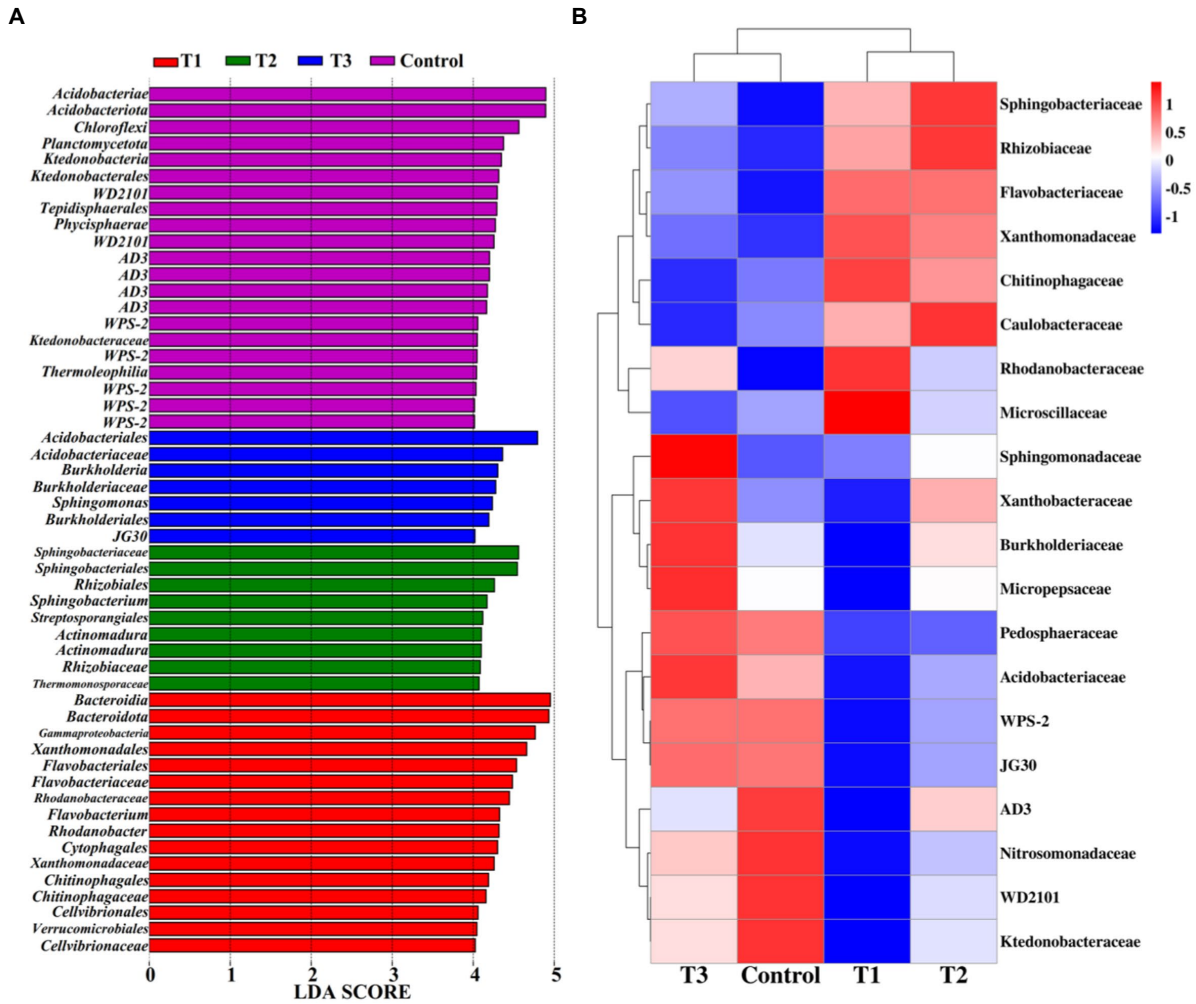
Linear discriminant analysis (LDA) effect size (LeFSe) of the bacterial taxa **(A)**, which identifies the most differentially abundant taxa among MF-HZ23 and CCF in different treatments. Only taxa with LDA values greater than 4 (*p* < 0.05) are shown. Hierarchical clustering analysis and heat map at the family level **(B)**. The tree plot represents a clustering analysis of the top 20 bacteria at family levels according to their Person correlation coefficient matrix and relative abundance, the upper tree plot represents a clustering analysis of soil samples according to the Euclidean distance of data. T1: MF-HZ23 at 3.00 kg/m^2^; T2: MF-HZ23 at 3.00 kg/m^2^ plus CCF at 0.02 kg/m^2^; T3: CCF at 0.04 kg/m^2^; and T4: control.

Linear discriminant analysis of effect size (LeFSe, LDA > 4, *p* < 0.05) was used to reveal the biomarkers with the largest difference between the rhizosphere soil bacterial communities of pakchoi under MF-HZ23 and CCF in different treatments (Figure 5A). A total of 53 bacterial biomarkers were found in MF-HZ23 (T1), MF-HZ23 plus CCF (T2), CCF (T3), and the control. In detail, MF-HZ23 (T1) is enriched with *Bacteroidia*, *Bacteroidota*, *Gammaproteobacteria*, *Xanthomonadales*, *Flavobacteriales*, *Flavobacteriaceae*, *Rhodanobacteraceae*, *Flavobacterium*, *Rhodanobacter*, *Cytophagales*, *Xanthomonadaceae*, *Chitinophagales*, *Chitinophagaceae*, *Cellvibrionales*, *Verrucomicrobiales*, and *Cellvibrionaceae*, MF-HZ23 plus CCF (T2) is enriched with *Sphingobacteriaceae*, *Sphingobacteriales*, *Rhizobiales*, *Sphingobacterium*, and *Streptosporangiales*, two types *Actinomadura*, *Rhizobiaceae*, and *Thermomonosporaceae*, CCF (T3) is enriched with *Acidobacteriales*, *Acidobacteriaceae*, *Burkholderia*, *Sphingomonas*, and *Burkholderiales*, *JG30*, the control is enriched with *Acidobacteriae*, *Acidobacteriota*, *Chloroflexi*, *Planctomycetota*, *Ktedonobacteria*, and *Ktedonobacterales*, two types *WD2101*, *Tepidisphaerales*, *Phycisphaerae*, four types *AD3*, five types *WPS-2*, *Ktedonobacteraceae*, and *Thermoleophilia*. Furthermore, the difference in relative abundance composition (family level) of the rhizosphere bacterial community under MF-HZ23 and CCF in different treatments was visually explained through heat maps (Figure 5B). MF-HZ23 (T1) was enriched with *Xanthomonadaceae*, *Chitinophagaceae*, *Rhodanobacteraceae*, and *Microscillaceae*, but was reduced with *Xanthobacteraceae*, *Burkholderiaceae*, *Micropepsaceae*, *Acidobacteriaceae*, *WPS-2*, *JG30*, *AD3*, *Nitrosomonadaceae*, *WD2101*, and *Ktedonobacteraceae* (*p* < 0.05). MF-HZ23 plus CCF (T2) was enriched with *Sphingobacteriaceae*, *Rhizobiaceae*, and *Caulobacteraceae*, but none was obviously reduced (*p* < 0.05). The results demonstrate that different fertilizer treatments such as MF-HZ23 (T1), MF-HZ23 plus CCF (T2), and CCF (T3) can increase or induce the existence of specific species, resulting in a change in the bacterial community structure in rhizosphere soil of pakchoi. In other words, those microbes may have greater potential to colonize and alter soil chemistry and microbial communities in immature soil.

### 3.6. The effect of MF-HZ23 on RDA of soil properties and microbial communities

Soil properties exhibited an influence in the composition of bacterial communities in pakchoi rhizosphere soil at the genus levels (Figure 3B; Table 4). Redundancy discriminant analysis (RDA) was carried out to examine the correlation between environmental factors and bacterial communities. Results from this study showed a total of 78.71% of the cumulative variance of the rhizosphere bacterial community-factor correction at the genus level (Figure 3B). Moreover, the *p* value displayed the significance of the relationship between the individual environmental factor and bacterial communities, and the values were 0.0025, 0.0025, 0.0050, 0.0005, and 0.0005 for pH, OMC, total N, available P, and AHN, respectively. Meanwhile, the contributions of the five main variables: available P, AHN, pH, OMC, and total N, explained 93.56, 89.63, 76.70, 76.16, and 72.62% of the bacterial community at the genus level, respectively. All those suggested that available P, AHN, pH, OMC, and total N were main factors influencing the bacterial communities (Table 4). Results also showed a complex relationship between bacterial growth and soil nutrient elements because the compositions of the bacterial communities in pakchoi rhizosphere soil were significantly affected by different soil properties.

**Table 4:** Contribution of soil environment to bacteria taxa at the genus level.

Soil environment	Contribution at bacterial genus level (%)
pH	76.16%
OMC	76.70%
Total N	72.62%
Available P	93.56%
AHN	89.63%

### 3.7. The effect of MF-HZ23 on rhizosphere soil metabolomics

**Figure 6:**
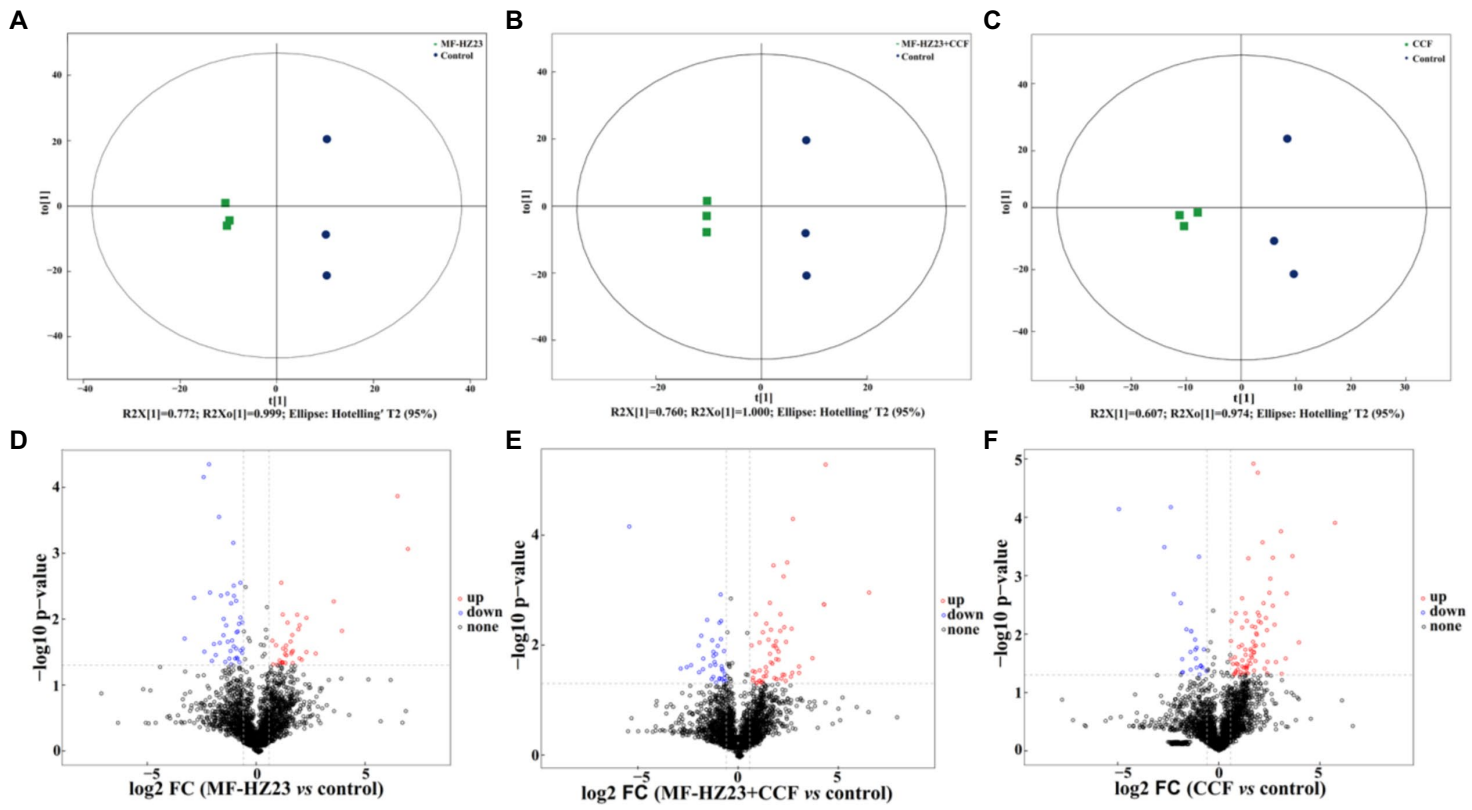
Orthogonal partial least squares-discriminant analysis (OPLS-DA) score map of pakchoi rhizosphere soil of the MF-HZ23 **(A)**, MF-HZ23 plus CCF **(B)**, and CCF treatment **(C)**. Volcano plot of differentially accumulated metabolites in MF-HZ23 vs. the control **(D)**, MF-HZ23 plus CCF vs. the control **(E)**, and CCF vs. the control **(F)**. Each point represents a metabolite, blue point indicates metabolities with FC ﹤ 0.67 and *p* ﹤ 0.05, red point indicates metabolities with FC ﹥ 1.5 and *p* ﹤ 0.05, black point indicates non-significant metabolites.

A score map of metabolites was also achieved by using the orthogonal partial least squares-discriminant analysis (OPLS-DA, a supervised statistical method of discriminant analysis; Figures 6A–C; Ren et al., 2021a). Results showed that the distribution of different treatments could be effectively separated between MF-HZ23 (T1), MF-HZ23 plus CCF (T2), CCF (T3), and the control. Indeed, Figure 6A presents the distribution of the sample points of the MF-HZ23 treatment and the control in the positive and negative area of *t*(1), respectively, while the model values of MF-HZ23 and the control were R^2^X(*cum*) = 0.772, R^2^Y(*cum*) = 0.999, and Q^2^(*cum*) = 0.750 (Q^2^ ﹥ 0.5). Similarly, Figure 6B presents the distribution of the sample points of the MF-HZ23 plus CCF treatment and the control in the positive and negative area of *t*(1), respectively, while the model values of MF-HZ23 plus CCF and the control were R^2^X(*cum*) = 0.760, R^2^Y(*cum*) = 1.000, Q^2^(*cum*) = 0.633 (Q^2^﹥0.5). Meanwhile, Figure 6C presents the distribution of the sample points of the CCF treatment and the control in the positive and negative area of *t*(1), respectively, while the model values of CCF and the control were R^2^X(*cum*) = 0.607, R^2^Y(*cum*) = 0.974, Q^2^(*cum*) = 0.372 (0.3﹤Q^2^ ≤ 0.5). Results showed that the interpretation and predication ability of the three groups of models were good due to that the Q^2^(*cum*) were greater than 0.3, especially MF-HZ23 and the control, MF-HZ23 plus CCF and the control groups (Q^2^(*cum*) greater than 0.5). Therefore, it can be inferred from the obvious separation of samples that the types of metabolites in the control rhizosphere soil were significantly changed by the application of the MF-HZ23, MF-HZ23 plus CCF, CCF, especially MF-HZ23 and MF-HZ23 plus CCF. Furthermore, volcano plot [based on fold change (FC) analysis] also showed that the metabolites in MF-HZ23, MF-HZ23 plus CCF, and CCF treatment were significantly different from the control (Figures 6D–F).

**Figure 7:**
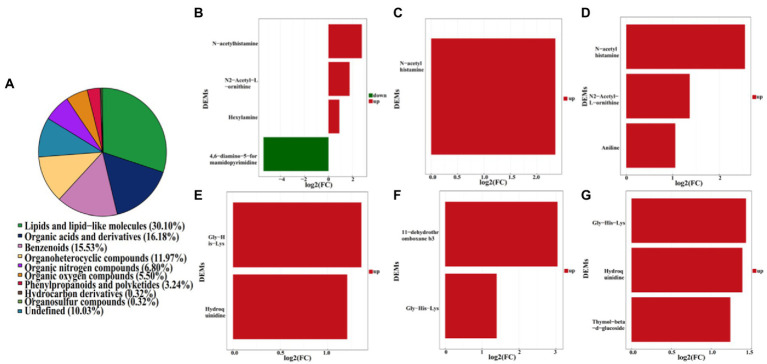
Metabolite classification statistics **(A)**, and DEMs in different treatment groups. **(B,E)** MF-HZ23 vs. the control; **(C,F)** MF-HZ23 plus CCF vs. the control; **(D,G)** CCF vs. the control. Red and green represents upregulated and downregulated, respectively.

Furthermore, a total of 4,417 pakchoi rhizosphere soil metabolites were screened in four different treatments, among which 309 metabolites were identified by using non-targeted metabolomics techniques (UHPLC–MS analysis). These metabolites mainly refer to lipids and lipid-like molecules (30.10%), organic acids and derivatives (16.18%), benzenoids (15.53%), organoheterocyclic compounds (11.97%), organic nitrogen compounds (6.80%), organic oxygen compounds (5.50%), phenylpropanoids and polyketides (3.24%), hydrocarbon derivatives (0.32%), organosulfur compounds (0.32%), and others undefined (10.03%; Figure 7A). DEMs among different treatment groups were selected with VIP (variable importance for the projection)﹥1 and *p* value﹤0.05. Results revealed that there were six DEMs belong to organoheterocyclic compounds, organic acids and derivatives, organic nitrogen compounds between MF-HZ23 and the control, with 4,6-diamino-5-formamidopyrimidine downregulated, and N-acetylhistamine, hexylamine, N2-acetyl-L-ornithine, gly-his-lys, and hydroquinidine upregulated; there were three DEMs belong to organic acids and derivatives, lipids and lipid-like molecules between MF-HZ23 plus CCF and the control, with N-acetylhistamine, 11-dehydrothromboxane b3, Gly-His-Lys up regulated; and there were six DEMs belong to organic acids and derivatives, benzenoids, lipids, and lipid-like molecules between CCF and the control, with N-acetylhistamine, aniline, N2-acetyl-L-ornithine up regulated (Figures 7B–G; Table 5). In detail, N-acetylhistamine and Gly-His-Lys were common metabolites in all three treatment groups; N2-acetyl-L-ornithine and hydroquinidine were common metabolites in MF-HZ23 and the control group and CCF and the control group; hexylamine and 4,6-diamino-5-formamidopyrimidine were unique metabolites in MF-HZ23 and the control group; 11-dehydrothromboxane b3 was unique metabolites in MF-HZ23 plus CCF and the control group; aniline and thymol-beta-d-glucoside were unique metabolites in CCF and the control group. All those results indicated that these metabolites might play a crucial role in the response of pakchoi to MF-HZ23 or CCF.

**Table 5:** List of differentially expressed metabolites (DEMs) by VIP, FC, and *p* value.

Compound name	Superclass	Treatment		Control	VIP	Log2 (FC)	*p* value	Regulated
N-acetylhistamine	Organic acids and derivatives	MF-HZ23	65,252,384	9428425.7	1.589	6.92	0.02	Up
Hexylamine	Organic nitrogen compounds		141,996,848	75,684,148	1.695	1.88	0.02	Up
N2-Acetyl-L-ornithine	Organic acids and derivatives		43,802,283	12,877,157	1.154	3.4	0.04	Up
4,6-diamino-5-formamidopyrimidine	Organoheterocyclic compounds		1690030.8	71,742,122	1.954	0.02	0	Down
Gly-His-Lys	Organic acids and derivatives		1.288E+09	502,041,316	1.843	2.57	0.03	Up
Hydroquinidine	-		1.589E+09	687,087,454	1.92	2.31	0.04	Up
N-acetylhistamine	Organic acids and derivatives	MF-HZ23+CCF	48,093,015	9428425.7	1.388	5.1	0.04	Up
11-dehydrothromboxane b3	Lipids and lipid-like molecules		81,869,303	9934347.2	1.275	8.24	0.03	Up
Gly-His-Lys	Organic acids and derivatives		1.317E+09	502,041,316	5.259	2.62	0.04	Up
N-acetylhistamine	Organic acids and derivatives	CCF	55,551,889	9428425.7	1.74	5.89	0.01	Up
Aniline	Benzenoids		131,149,149	63,007,559	1.945	2.08	0.01	Up
N2-Acetyl-L-ornithine	Organic acids and derivatives		33,197,404	12,877,157	1.108	2.58	0.02	Up
Hydroquinidine	-		1.8E+09	687,087,454	4.64	2.62	0.03	Up
Thymol-beta-d-glucoside	Lipids and lipid-like molecules		833,742,930	353,486,475	2.995	2.36	0.04	Up
Gly-His-Lys	Organic acids and derivatives		1.357E+09	502,041,316	4.108	2.7	0.05	Up

**Figure 8:**
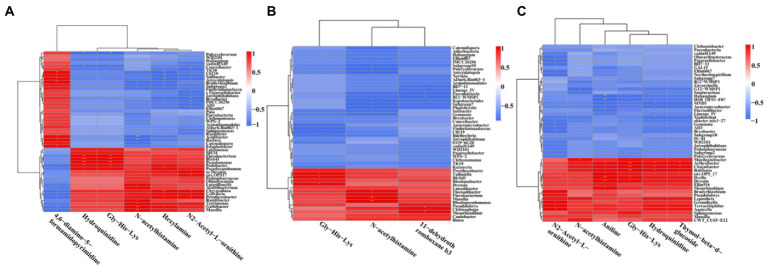
Correlation heat map between DEMs and related bacteria in different treatment groups. **(A)** MF-HZ23 vs. the control; **(B)** MF-HZ23 plus CCF vs. the control; and **(C)** CCF vs. the control. Red and blue represent positive correlation and negative correlation, respectively. * indicated a significant correlation at *p* < 0.05.

**Figure 9:**
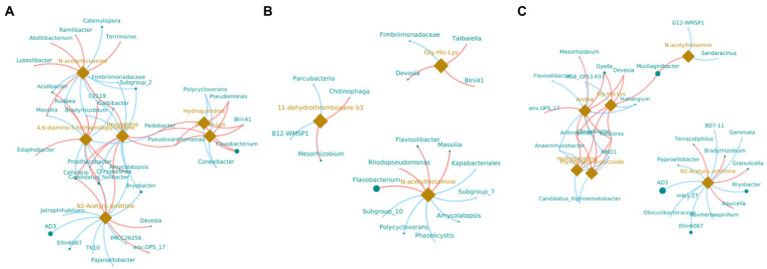
Connection network between DEMs and related bacteria in different treatment groups. **(A)** MF-HZ23 vs. the control; **(B)** MF-HZ23 plus CCF vs. the control; and **(C)** CCF vs. the control. Green dots and orange prisms represent bacteria and metabolites, and the size of nodes and prisms represents the relative abundance of bacteria and metabolites, respectively. Red lines and blue lines represent positive correlation and negative correlation, respectively.

In order to understand the correlation of bacteria with metabolites, the metabolites with significant differences were normalized, and the clustering heat map and connection network were drawn (Figures 8, 9). Results indicated that in MF-HZ23 and the control group, six DEMs were significantly positively correlated with 23 genus of bacteria, among which N-acetylhistamine was positively correlated with *Massilia*, *Galbibacter*; *Luteolibacter*, *Abditibacterium*, *Ramlibacter*, and *Terrimonas*; Hexylamine was positively correlated with *Chryseolinea*, *Pedobacter*, *Pseudoxanthomonsa*, *Cellvibrio*, *Prosthecobacter*, and *Galbibacter*; N2-acetyl-L-ornithine was positively correlated with *Devosia*, *env.OPS17*, *Chryseolinea*, *Cellvibrio*, and *Prosthecobacter*; Gly-His-Lys and Hydroquinidine were positively correlated with *Blrii41*, *Pseudomonas*, *Flavobacterium*, *Pedobacter*, and *Prosthecobacter*; 4,6-diamino-5-formamidopyrimidine was positively correlated with *Bradyrhizobium*, *C0119*, *Amycolatopsis*, *Acidibacter*, *Rudaea*, and *Edaphobacter*. In MF-HZ23 plus CCF and the control group, three DEMs were significantly positively correlated with nine genus of bacteria, among which N-acetylhistamine was positively correlated with *Massilia*, *Rhodopseudomonas*, *Flavobacterium*, and *Flavisolibacter*; 11-dehydrothromboxane b3 was positively correlated with *Chitinophaga* and *Mesorhizobium*; Gly-His-Lys was positively correlated with *Taibaiella*, *Blrii41*, and *Devosia*. In CCF and the control group, six DEMs were significantly positively correlated with 12 genus of bacteria, among which N-acetylhistamine was positively correlated with *Mucilaginibacter*; Aniline was positively correlated with *Dyella*, *Deyosia*, *Arthrobacter*, *Chujaibacter*, *Ralstonia*, *env.OPS17*, and *Mesorhizobium*; N2-Acetyl-L-ornithine was positively correlated with *Bradyrhizobium*, *Granulicella*, *Terracidiphilus*, and *Aquicella*; Hydroquinidine and Thymol-beta-d-glucoside was positively correlated with *Arthrobacter*, *Chujaibacter* and *Ralstonia*; Gly-His-Lys was positively correlated with *Arthrobacter*, *Chujaibacter*, *Ralstonia*, *Mucilaginibacter*, *Dyella*, and *Deyosia*. In general, the DEMs (organic acids, lipid, and secondary metabolites) by the application of different fertilizers especially MF-HZ23 in the rhizosphere soil of pakchoi may enrich the soil bacteria and help coordinate the rhizosphere bacteria.

## 4. Discussion

Land reclamation is regarded as a good way to keep balance of occupation and compensation. However, immature soil is not suitable for plant growth. In most cases, the production capacity of newly reclaimed land is only 10–30% of the occupied cultivated land in China (Wang et al., 2017). In practice, immature soil can be modified by adding organic matter and PGP microbes to promote soil maturity and plant growth (Larney and Angers, 2012; Rashid et al., 2016; Li et al., 2021a). Over the last few years, the continued application of organic amendments, such as planting winter legume crops, promoting the use of commercial organic fertilizer, utilizing animal manure, was carried out to improve the soil quality of newly reclaimed land (Drozdowski et al., 2012; Watkinson et al., 2017; Teixeira et al., 2019). Recently, PGPF-based biofertilizers have attracted significant attention due to the environment friendly and agriculture sustainability abilities (Egamberdieva and Adesemoye, 2016; Hossain and Sultana, 2020). Studies showed that the effect of PGPF was largely dependent on their successful colonization, growth, and efficient adaptation to rapidly changing conditions (Wakelin et al., 2007; Araujo et al., 2020; Li et al., 2021b). Compared to the large number of PGPF identified in the laboratory, only a small fraction of them was applied in field condition (Hossain and Sultana, 2020). Whereas development of appropriate application methods could improve the performance of PGPF in the field (Lyn et al., 2010), including protecting biological actives from stress, ensuring viability and microbial actives in the field (Lyn et al., 2010). In the literature, several studies have reported the pig corpses, corn and sugarcane bagasse was potential carriers for PGPF (Doni et al., 2014; Oancea et al., 2016). In our study, sheep manure and mushroom residue was choosen to carry *A. brunneoviolaceus* HZ23, and the effect of microbial fertilizer based *A. brunneoviolaceus* HZ23 (MF-HZ23) on packchoi growth, soil properties, rhizosphere bacterial community structure, and metabolites in newly reclaimed land was explored systematically at the very first time.

In our recent study, HZ23 has been reported to cause an obvious increase in leaf size, root and seedlings length, fresh weight, and dry weight of eggplants compared to the controls on newly reclaimed land (Li et al., 2021b). As we known, the effect of PGPF is largely depending on their successful survival, colonization, growth, and efficient adaptation to environmental conditions. HZ23 may have great potential to colonize in immature soil, and have the capacity to enhance the solubilization of insoluble phosphate compounds, produce siderophore and IAA (Li et al., 2021b). Similarly, MF-HZ23 generally had a greater effect on pakchoi growth compared to CCF and the control in our study. Moreover, some previous studies also reported that the use of microbial fertilizer could improve the production of crop. For example, Zhang et al. (2018) showed that yield of facility tomato was improved after the application of microbial fertilizers (especially ABA-2). Li et al. (2022c) reported that the application of Tuzangjin microbial fertilizer, carbonergic microbial agent, and seaweed fertilizer resulted in a greater promotion effect on corn production in newly reclaimed land, and the applied amount of microbial fertilizers could be reduced as the soil fertility improves.

The soil properties in the packchoi field were differentially affected by different treatments, and the effect was dependent on the soil parameters and the kind of fertilizer. Indeed, results showed that the soil pH and OMC can be improved by microbial fertilizer (MF-HZ23, T1, and T2), and reduced by CCF (T3), and similar observation was observed by Li et al. (2021a, 2022c), which may be due to the proportion of microaggregates (<0.25 mm) of soil decreased by heavy use of CCF. Previous studies revealed the fertility and quality of immature soil could be improved by enhancing the low pH and OMC, which have been regarded as the basis for soil fertility and quality (Li et al., 2022a,b). As well known, planting winter legume crops, application of commercial organic fertilizer, utilizing farmyard manure and crop straw, and application of microbial fertilizer can not only increase crop yield but also can maintain a sustainable increasing trend in crop yield owing to the improvement of soil fertility (Asghar and Kataoka, 2022), and the OMC has been proposed to exhibit a greatest effect on microbial communities than the other soil parameters (Baran et al., 2019; Qu et al., 2019). Results also showed that the application of microbial fertilizer (MF-HZ23, T1, and T2) resulted in the greater increase in all the other nutrient elements than that of CCF (T3) and the control. In particular, MF-HZ23 (T1) and MF-HZ23 plus CCF (T2) caused significantly increase in available P. The available P of MF-HZ23 (T1), MF-HZ23 plus CCF (T2) were 854.09 and 498.30 mg/kg, respectively, greater than that of CCF (T3) and the control (48.99 and 38.82 mg/kg, respectively). Li et al. (2015) showed that large areas of Chinese arable land with Olsen *p* values were lower than 20 mg/kg (the critical fertility level for most field crops), and the great challenge for P management in China was to improve the fertility of low-P soils, increase P use efficiency, and minimize the risk of environmental P losses (only 20% of fertilizer P could be used by crops during the growing season in China). As we know, the pH of soil could be reduced by CCF, whereas the main P minerals become more soluble assoil pH increases (especially, variscite, strengite, and calcium P become more soluble in pH 6–6.5; Li et al., 2015). In our study, the soil pH of MF-HZ23 (T1), MF-HZ23 plus CCF (T2; 6.35 and 5.35, respectively) was higher than CCF (T3) and the control (4.55 and 4.86, respectively). Thus, compared to CCF and the control, the pH of MF-HZ23 (T1), MF-HZ23 plus CCF (T2) was more friendly to HZ23 to solubilize P, especially MF-HZ23 (T1). Therefore, it can be inferred that MF-HZ23 may be able to have great effect in improvement of newly reclaimed soil.

We measured the bacterial community diversity in the packchoi rhizosphere with different fertilizers (MF-HZ23, MF-HZ23 plus CCF, CCF, and the control) using 16S rRNA gene high-throughput sequencing. High microbial diversity promotes soil ecosystem functioning (Maron et al., 2018). We used the number of ASVs and the alpha diversity (Chao1 and Shannon index) to measure the bacterial diversity under different fertilizer treatment conditions (Figures 2A–C). Results showed that the bacterial ASVs number was significantly increased by MF-HZ23, and MF-HZ23 increased the Chao1 index but had no significant effect on the Shannon index. We considered that MF-HZ23 had the obvious effect on increasing the richness of bacterial communities in short term (55 days), and it should be worth to elucidate the effect of MF-HZ23 on the diversity of bacterial communities in long term in future. The role of different fertilizer in soil microbe has been reported in previous studies. For example, 3 consecutive years application of three kinds of microbial fertilizer (CMA, TMF, and SMF) could increase the number of OTUs and Chao1 index of the bacterial community of corn rhizosphere soil in newly reclaimed land, but no significant difference was observed in the Shannon index (Li et al., 2022c). Two kinds of commercial organic fertilizer, including PMMR-OF and SM-OF, could cause changes in the bacterial OTUs, Chao1, and Shannon index of corn rhizosphere soil (Li et al., 2022a). The bacterial OTUs and Chao1 index of sweet potato rhizosphere soil in newly reclaimed land was reduced by CCF (Li et al., 2022b). Applying bio-organic fertilizer in combination with 30% chemical fertilizer could increase the number of bacteria species in the community (Jiang et al., 2019). According to previous studies, the soil fertility is associated with the richness of some special microbes, which can mediate nutrient mobilization through nitrogen-fixing, solubilization inorganic P, producing IAA, and siderophore to promote plant growth (Plassard et al., 2011; Ahemad and Kibret, 2014; Mendes et al., 2014; Ikram et al., 2018). In other words, the improvement of the soil quality by different fertilizer (for instance, MF-HZ23) may be partially attributed to the enrichment of specific microbes. Further study was carried out to elucidate the role of specific microbes in the fertility improvement in immature soil. PCA results showed that the rhizosphere bacterial communities were well separated according to different fertilizer treatments (Figure 3A, PERMANOVA, *p* < 0.05), and the different fertilizer explained 26.5% (*p* = 0.001) of the variation. Therefore, there were significant differences in bacterial community composition between different fertilizer treatments. In accordance with the results of this study, continuous application of commercial organic fertilizer (COF, MR) in immature soil caused a significant change in bacterial communities in sweet potato soil (Li et al., 2022b); the application of four different fertilization regimes (100% bio-OF, 70% bio-OF +30% *CF*, 30% bio-OF +70% *CF*, and 100% *CF*) caused a significant change in the PCA of the bacterial communities in tomato coastal saline soil (Jiang et al., 2019). Taken overall, the application of MF-HZ23 could reshape the rhizosphere bacterial community of packchoi in newly reclaimed land.

At the phylum level, all different treatments included *Protebacteria*, *Bacteroidota*, *Acidobacteriota*, *Actinobacteriota*, *Chloroflexi*, *Planctomycetota*, *Verrucomicrobiota*, *Patescibacteria*, and *Myxococcota* (Figure 4A), while at the species level, all different treatments included *Sphingomonas*, *WD2101*, *Burkholderia*, and *Mucilaginibacter* in four species (Figure 4B). The change in the number of specific bacteria in different treatments may be due to the different nutrients between microbial and chemical fertilizers. Indeed, more attention should be paid to *Sphingomonas*, *Burkholderia*, and *Mucilaginibacter*. *Sphingomonas* is reported to play a role in plant growth by producing plant growth hormones during salinity, drought, and heavy metal stress conditions in agricultural soil (Jiang et al., 2016). *Burkholderia* has been reported to have a great potential in promoting the growth of eggplants in immature soil by phosphate solubilization, nitrogen fixation, siderophore production, and indole acetic acid production (Li et al., 2021c). *Mucilaginibacte* not only have the ability to hydrolyze organic matter, such as pectin, xylan, and laminarin, produce large amount of extracellular polysaccharides containing the sugars glucose, mannose, and galactose, and but also can promote cotton growth (Pankratov et al., 2007; Urai et al., 2008; Madhaiyan et al., 2010; Han et al., 2012). Our previous studies also showed that the application of organic or microbial fertilizer in immature soil could improve the soil quality, which may be due to the change in the genera abundance distribution by enriching specific microbe in soil (Li et al., 2022a,b,c).To further explore the impact of MF-HZ23 on bacterial groups, we used liner discriminant analysis of the effect size (LeFSe, LDA > 4, *p* < 0.05), and a total of 53 bacterial biomarkers were got in four different treatments. Furthermore, heat map indicated that MF-HZ23 could significantly enrich *Xanthomonadaceae*, *Chitinophagaceae*, *Rhodanobacteraceae*, *Microscillaceae*, *Sphingobacteriaceae*, *Rhizobiaceae*, and *Caulobacteraceae*. It is worth noting that some species of *Xanthomonadaceae*, *Chitinophagaceae*, *Sphingobacteriaceae*, and *Rhizobiaceae* play an important role in enhancing resilience to plant pathogens and promoting plant growth (Jiang et al., 2016; Luo et al., 2019). Previous studies also showed that microbial fertilizers, including Tuzangjin microbial fertilizer, carbonergic microbial agent, and seaweed microbial fertilizer, could improve the soil fertility by changing the microbial community structure in rhizosphere soil (Li et al., 2022c). In addition, we have also isolated several fungal and bacterial strains from new reclamation soil, which have great ability to solubilize phosphate, fix nitrogen, and produce siderophores and indole acetic acid in immature soil (Li et al., 2021b,c). In other words, those microbes may have greater potential to colonize and alter soil chemistry and microbial communities in immature soil.

A total of 4,417 metabolites were screened from pakchoi rhizosphere soil in different treatments. Results of OPLS-DA showed that metabolites were significantly changed by MF-HZ23, which was also verified by volcano plot (Figure 6). A total of 309 metabolites were identified by UHPLC–MS analysis, which mainly belong to lipids and lipid-like molecules (30.10%), organic acids and derivatives (16.18%), benzenoids (15.53%), and organoheterocyclic compounds (11.97%; Figure 7A). As we known, organic acids, lipids, and other secondary metabolites are the essential biological sources for soil bacteria rhizosphere colonization, growth, and symbiosis; they also play an important role in plant root growth, including energy storage and regulating cell signaling and ion transport (Sonnino and Prinetti, 2013; Lucie et al., 2016). To further explore the correlation of bacteria with DEMs, the clustering heat map and connection network were drawn (Figures 8, 9). Results showed that there were six DEMs in MF-HZ23 and the control group, with five DEMs upregulated and one DEM downregulated (Figures 7B,E; Table 5). And those six DEMs were significantly positively correlated with 23 genus of bacteria, which showed complicated interactions between bacteria and DEMs in pakchoi rhizosphere soil.

## 5. Conclusion

In summary, our results showed that MF-HZ23 had a great effect on the growth of pakchoi, and also had great effect on improving soil pH, OMC, total N, AHN, and available P. This suggests that HZ23 can be a good plant promoting microbe, and sheep manure and mushroom residue was good carriers for HZ23 used in newly reclaimed land. Compared with the control, MF-HZ23 caused a differential change in bacterial community of the newly reclaimed land. Some specific plant growth promoting bacteria, such as *Sphingomonas*, *Burkholderia*, *Mucilaginibacter*, *Xanthomonadaceae*, *Chitinophagaceae*, *Sphingobacteriaceae*, and *Rhizobiaceae*, have been found to be closely related to the soil improvement by MF-HZ23, indicating the relationships among bacteria, micro-fertilizer, and soil. Furthermore, the composition of bacterial communities in packchoi rhizosphere soil was affected significantly by avaiable P, AHN, pH, OMC, and total N at the genus level. In particular, MF-HZ23 resulted in a great change in the kinds and the relative contents of metabolites in the rhizosphere soil. Correlation heat map and connection network revealed a significant correlation at the genus levels between related bacterial groups and DEMs of packchoi rhizosphere soil of MF-HZ23. All the above results revealed that MF-HZ23 could improve packchoi growth, and influence the interaction among soil properties, bacterial community, and secondary metabolites. Overall, the result of this study clearly revealed that MF-HZ23 played an important role in improving the soil quality and pakchoi production in newly reclaimed land.

## Data availability statement

The original contributions presented in the study are included in the article/supplementary material; further inquiries can be directed to the corresponding authors.

## Author contributions

XL, QL, JY, and BL: conceptualization and writing–original draft preparation. XL, DL, XR, HW, TA, and BL: methodology. XL, DL, and TA: software. XL, DL, XR, and JY: validation. DL, YZ, HW, and TA: formal analysis. XL, YJ, JX, and XR: investigation. XL, JY, XR, QL, and BL: resources. QL and JY: data curation, project administration, and funding acquisition. JY, QL, BL, and KG: writing—review and editing and supervision. XL, JY, QL, BL, and KG: visualization. All authors contributed to the article and approved the submitted version.

## Funding

This research was funded by the Key Research and Development Program of Zhejiang Province (2019C02035), the Science and Technology Innovation and Promotion Demonstration project of Hangzhou Academy of Agricultural Sciences (2022HNCT-13, 2022HNCT-07, and 2022HNCT-02), and the National Natural Science Foundation of China (31872017 and 32072472).

## Conflict of interest

The authors declare that the research was conducted in the absence of any commercial or financial relationships that could be construed as a potential conflict of interest.

## Publisher’s note

All claims expressed in this article are solely those of the authors and do not necessarily represent those of their affiliated organizations, or those of the publisher, the editors and the reviewers. Any product that may be evaluated in this article, or claim that may be made by its manufacturer, is not guaranteed or endorsed by the publisher.
